# Designing Smart Biomaterials for Tissue Engineering

**DOI:** 10.3390/ijms19010017

**Published:** 2017-12-21

**Authors:** Ferdous Khan, Masaru Tanaka

**Affiliations:** 1 Soft-Materials Chemistry, Institute for Materials Chemistry and Engineering, Kyushu University, 744 Motooka Nishi-ku, Fukuoka 819-0395, Japan; 2 Frontier Center for Organic Materials, Yamagata University, 4-3-16 Jonan, Yonezawa, Yamagata 992-8510, Japan

**Keywords:** tissue engineering, smart materials, extracellular matrix, stimuli responsive polymer

## Abstract

The engineering of human tissues to cure diseases is an interdisciplinary and a very attractive field of research both in academia and the biotechnology industrial sector. Three-dimensional (3D) biomaterial scaffolds can play a critical role in the development of new tissue morphogenesis via interacting with human cells. Although simple polymeric biomaterials can provide mechanical and physical properties required for tissue development, insufficient biomimetic property and lack of interactions with human progenitor cells remain problematic for the promotion of functional tissue formation. Therefore, the developments of advanced functional biomaterials that respond to stimulus could be the next choice to generate smart 3D biomimetic scaffolds, actively interacting with human stem cells and progenitors along with structural integrity to form functional tissue within a short period. To date, smart biomaterials are designed to interact with biological systems for a wide range of biomedical applications, from the delivery of bioactive molecules and cell adhesion mediators to cellular functioning for the engineering of functional tissues to treat diseases.

## 1. Introduction

Research on polymer biomaterials has been a subject of interest both in academia and industry since 1960 [1]. The variety of polymeric materials has been rationally designed by incorporating distinct functional groups into the molecular chain to control physical, chemical, and biological properties for versatile biomedical arenas, such as the controlled delivery of bioactive molecules and cell-based therapeutic applications [2,3,4]. However, the nature of the biomedical applications of such material systems depends on their macromolecular structure, their interactions with living cells and cytocompatibility, and how the macromolecules organize by themselves to form three-dimensional (3D) architecture. Polymer-based biomaterials have been developed both from natural and synthetic (man-made) resources. Polysaccharides and proteins are well–established natural polymeric biomaterials, which have found numerous applications in tissue regeneration. For example, chitosan is one of the best polysaccharides with exceptional biocompatibility and biodegradability, consisting of multifunctional groups that make it a potential candidate for future biomaterials development (via processing) for cellular functioning and differentiation towards tissue engineering [5]. Protein-based biomaterials, for example, collagen, metrigel, and hyaluronic acid, have been derived from animal sources and explored in tissue regeneration. Although proteins are highly biocompatible, their very fast degradation and low mechanical strength result in a lack of structural support during tissue development. Polymeric biomaterials are also developed by polymerizing one or more monomers either by homopolymer reaction or copolymer reaction to form non-biodegradable [6,7] and biodegradable materials [8,9]. The structure, molecular chain length, and stereochemistry can be tailored by varying chemical and physical parameters during synthesis. In recent years, a significant demand for biodegradable materials development, by means of synthesis, in addition to their processing and fabrication of 3D structural scaffolds has arisen in tissue regeneration and the delivery of bioactive molecules, due to the demand for cost-effective surgical procedures. Thus, continuous efforts need to be undertaken in this field.

For biomedical applications, biomaterial scientists have devoted continuous efforts to developing methods, fabricating new medical devices, and processing and synthesizing novel biomaterials. Recently, Liu and co-workers [10] developed a alginate-collagen 3D hydrogel composite, which has improved the treatment of corneal diseases. We developed high-throughput technology for cell-compatible biomaterials discovery, as well as the processing and fabrication of 3D scaffolds for potential application in skeletal tissue regeneration [11,12]. To date, a wide range of biomaterials have been used for biomedical applications, many of which do not have biomimetic properties, and require the engineering of these materials in a cost-effective manner for maximum output. Therefore, there is a socioeconomic need to develop new biomaterials for resolving such problems in the biomedical arena. Polymers with hydrophilic properties form 3D crosslinked networks, known as hydrogels, a type of novel smart biomaterial that responds to external stimulus and exhibits potential application as a scaffold for tissue regeneration and the delivery of bioactive molecules [10,13,14].

For tissue engineering (TE), a biomaterial scaffold provides mechanical and porous network structural support, shape, and hierarchy architecture with surface chemistry for cell attachment, cell-cell communication through the porous network, as well as proliferation and differentiation for tissue regeneration. To date, most synthetic biomaterials are derived for TE application, which are synthesized either from lactic acid, caprolactone, or glycolide monomers to form poly(l-Lactide), or poly(ε-caprolactone), or poly(l-glycolic acid), respectively, and/or their combination to form copolymers, or the physical blending of these polymers [11,12]. Natural polymers such as chitosan, alginate, starch, collagen, hyalauronic acid, cellulose, fibrin, silk, and their derivatives are also used [5,15,16]. A variety of techniques and methods has been developed to fabricate biomaterial scaffolds to control shapes, sizes, porosities, and architectures for TE applications [17,18]. In any tissue regeneration, it is important that biomaterial scaffolds not only provide temporary structural integrity but also play a role in the interaction of the cells and biomolecules, cell attachment and growth, as well as in the process of tissue development. However, poly(l-Lactide) and poly(ε-caprolactone) are known to be biodegradable materials, which have been implanted to identify their host and tissue regeneration [19,20]. However, such traditional biomaterials do not have the ability to adapt to living tissues during changing pH and body temperature caused by disease. Therefore, polymer scientists have been trying to create smart polymeric biomaterials [21] that mimic living tissues in the last two decades.

It is well known that the properties of smart polymers change reversibly with the sliding variation of external or internal parameters in the system, such as temperature, pH, ionic factor, biological molecules, and so on. Thus, smart polymeric biomaterials, in particular as delivery systems of bioactive molecules; tissue engineering scaffolds; and cellular attachment and growth have become novel research topics.

## 2. Tissue Engineering

**Figure 1:**
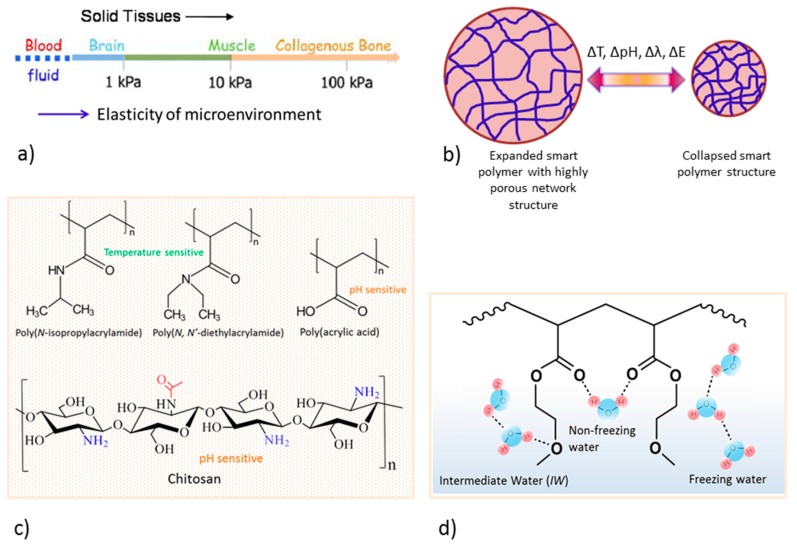
Smart polymer biomaterials for tissue engineering applications: (**a**) Representation of elastic modulus of various tissues in consideration to design a smart scaffold; (**b**) stimuli responsive system (∆T: variation of temperature change, ∆pH: variation of pH, ∆λ: variation of wave length, and ∆E: variation of electric field); (**c**) structures of some synthetic and natural smart polymers; and (**d**) novel scaffold of poly(2-methoxyethyl acrylate) (PMEA)—hydrated PMEA forms intermediate water, which affects the protein adsorption, as well as cell adhesion, proliferation, and differentiation.

The aim of TE is to develop new functional tissue and regenerate tissue either in vitro or in vivo to cure diseases when a surgical intervention is needed. In such circumstances, 3D biomaterial scaffolds play a critical role in repairing injury. For optimal functional tissue development, the scaffold should interact with cells without any adverse effect to provide cellular attachment, proliferation, growth, and accumulation of mineral matrix. In addition, it is essential to provide structural support, a design akin to the natural extracellular matrix, and a suitable surface, porosity, and heterogeneous pore sizes to promote cell-cell communication and differentiation, as well as transport nutrients. The 3D scaffolds should comply with sufficient mechanical strength similar to that of native tissue and a crosslinked network structure, as demonstrated in Figure 1a,b.

The 3D structured biomaterials can be designed utilizing a variety of natural and synthetic polymers (examples are presented in Figure 1c,d), ceramics such as hydroxyl apatite (HAp), and tricalcium phosphate (TCP), as well as their combinations to mimic native tissue while maintaining cell viability and functions. Furthermore, 3D scaffolds should act as a delivery agent for bioactive molecules or drugs and can be encapsulated into the materials during their process and fabrication for faster curing, if needed. The design and selection of smart biomaterials depends on their specific application, some of which are more suitable than others. Biomaterials can have a solid or hydrogel structure before implantation, or be in injectable forms that harden in situ.

### Extracellular Matrix (ECM)

The biological ECM provides structural support along with all necessary biological functions during tissue regeneration and maintenance. The scaffolds used in TE are also expected to have the same level of support and functional capability as that of the biological ECM [21]. ECM is a dynamic and complex structure, directly involved in specific gene regulation for a particular tissue development [22,23], and actively interacting with cells to remodel tissue [24]. The structure and components of ECM are continuously changing due to its dynamic nature with the development of tissues, remodeling, and repairing. It is critically important to develop smart and biomimetic 3D structure biomaterials that closely match the characteristic nature of the native ECM [25]. However, understanding complex functions and the structure of ECM in mature and/or during tissue development are extremely difficult. Combining multiple approaches, biomaterial scientists have developed a 3D structure with close approximation of natural ECM, and this has remained an active research area.

Several types of animal-derived proteins (e.g., collagen, laminin, and metrigel) have been utilized to fabricate 3D scaffolds for various TE, such as skin replacement, bone substitutes, artificial blood vessels [26], and cell delivery [27]. Hydrogels have been synthesized by the modification of polysaccharides via crosslinking for both drug release and tissue regeneration [28,29]. Hyaluronic acid (HA) is a polysaccharide consisting of linear glycosaminoglycan, found in the ECM and known to influence cell signaling pathways; it plays a crucial role in functional TE [29]. HA is the Food and Drug Administration (FDA) approved biomaterial for human application, and clinical trials confirmed that HAs are effective for osteoarthritis applications [30,31,32]. Chitosan (Figure 1c) is an important class of polysaccharide consisting of multiple functional groups, allowing ease of chemical modification, and has been used as a delivery agent, as well as in cell encapsulation, cellular functioning, and differentiation toward TE application [5,16,33,34]. Alginate is another example of a polysaccharide that has been used for cell encapsulation and injectable gel in TE [35].

## 3. Designing Smart Biomaterials

Designing bio-functional materials is critically important for potential biomedical applications. There are several approaches in the design of hybrid smart biomaterials, composed of synthetic and natural polymers, that considerably enhance the potential applications of such materials. These techniques permit the insertion of bio-recognition moieties into the structure of polymers that influence their self-assembly into precisely defined 3D structure formations. The design of smart polymer-based biomaterials with desired properties and network structure mainly depends on the characteristic nature of functional monomers and their feed ratio, method of polymerization and kinetics, building of molecular architecture during synthesis, and crosslink network formation. The development of advanced functional biomaterials requires control over some physical and chemical parameters in the design, synthesis, processing, and fabrication. The 3D network structure smart material mainly depends on the characteristic nature of the polymers, monomers, oligomers, and the methods of synthesis. There is a wide variety of methods which include radiation-induced crosslinking, chemical and physical crosslinking, for the preparation of 3D network biomaterials, namely hydrogels with different structures and properties. The hydrogel is not soluble in water, due to the presence of a 3D polymer network, and swells at equilibrium. The 3D networks in hydrogels are formed either by covalent bonding between polymer chains or physical interactions such as hydrogen boning and ionic bonding. The crosslinked network provides an equilibrium state between dispersing and cohesive forces between polymer chains, resulting in the insolubility of the gel in water. These materials have versatile applications as biomaterials, scaffolds for functional tissue regeneration, delivery agents, and medical devices.

Key factors that can influence the swelling of the 3D network of smart materials include: (i) the positive change of thermal energy during mixing of a polymer and solvent favors swelling; and (ii) the change of thermal energy is related to the polymer chain conformations, and negative thermal change prevents swelling.

These polymer networks can be classified as to their preparation methods, such as chemical reactions and physical blending. The examples of the chemical reactions of hydrogels are from the graft-copolymer [36], block copolymer [37], and crosslinking reactions of polymers [38]. For the hydrogel synthesis, the reactions can be initiated either by chemical initiation methods [38] or ionizing radiation-induced methods [39]. The chemical initiation methods require the incorporation of a bifunctional reagent and chemical catalyst, thus introducing impurities into the final product. Sometimes it can be extremely difficult to remove impurities from the hydrogel networks. Alternatively, the ionizing radiation-induced methods offer advantages that allow efficient crosslinking and yield a much cleaner product as compared to those of the chemical initiation methods. High-energy (e.g., γ-radiation, electron beam) radiation, plasma-radiation, and UV-radiation induced methods can be adopted to synthesize polymer hydrogels.

The physical blending of different polymers processed either by solution or thermally is an alternative approach and an economically viable route for the development of new structural smart materials [40], although it retains the characteristic properties of each polymer in the blend. There are several advantages to this system, including the ease of controlling the processing and fabrication of smart devices. This approach would allow achieving desired properties such as porosity, pore sizes, swelling ratio, biodegradability, and mechanical property by changing the process parameters, feed ratios of starting polymers, and selection of appropriate solvent. Therapeutic molecules can easily be incorporated into the blended solution or dispersed in micro-phase reservoirs.

Scientists are capable of designing and synthesizing polymeric biomaterials with complex architecture, by copolymerizing multiple chemical functional building blocks and tailoring biocompatibility, biodegradation, mechanical properties, hydrophilicity, and chemical and biological response to external stimuli, in a well-controlled manner. In such biomaterial system, the copolymer chains have the ability to organize by themselves in the water phase through intra- and intermolecular interactions, thus forming structural diversity. There are experimental challenges to designing model copolymer systems that will allow a faster response with slight variation of multiple stimuli. Despite the challenges of fabricating smart materials that respond to multiple stimuli (e.g., temperature, pH, and light), they have biomedical industrial importance. In the future, material scientists will continue to investigate such systems to achieve scientific breakthroughs.

In a broader aspect, smart biomaterials can be designed by incorporating peptide and/or protein into the polymer network, enabling the creation of a 3D scaffold for TE application [41,42]. For example, Ito and co-workers [42] developed smart biomaterials by genetically engineered collagen-type II protein, which was used for 3D scaffold fabrication to tailor chondrocytes proliferation and migration, and promoted artificial cartilage formation [42]. In a separate study, it was reported that cysteine-tagged fibronectin coupled with poly(ethylene glycol) (PEG) derivatives by employing Michael-type addition, then crosslinked with thiol-modified HA [43], formed a 3D hydrogel matrix. They demonstrated that these hydrogels were cytocompatible, fully supported human fibroblasts adhesion, proliferation, and robust migration, and after implantation in porcine cutaneous promoted dermal wound healing [39]. Similarly, a number of elastin-based block copolymers exhibiting a wide range of mechanical properties have been developed for TE application [44]. The signaling molecules in scaffold materials can be chemically linked to enhance cell binding. The functionalization of synthetic polymers by PNIPAm-(Arg-Gly-Asp (RGD)) peptide promotes cells attachment, migration, and functionality, and has been well reviewed [45].

In recent years, self-assembly systems have been developed both by chemically and biologically synthesized peptides for neural tissue engineering [46] and the growth of human dermal fibroblasts with excellent biocompatibility [47,48]. In the development of new biomaterials, self-assembly peptides and proteins are currently and will remain an active field of research, as this system responds to the external environment, namely, pH and temperature [49]. Although the generation of 3D-structure scaffolds by using self-assembly peptides is highly reproducible, it faces a number of challenges such as scale-up the process, production, and purification in a cost-effective manner. Without a doubt, enormous effort is required, focusing on the rational design, discovery, fabrication, and the process development.

With a deeper understanding of the fundamental behavior of biological complexity and the physicochemical nature of native tissue, the rational design of new smart biomaterials and production is made possible by applying multidisciplinary approaches, bringing together polymer chemists, biomaterials scientists, tissue engineers, and medical surgeons.

## 4. Importance of Smart Biomaterials in TE

There is a socioeconomic need to replace and/or repair tissue with more advanced materials approach, techniques, and methods focusing on functional tissue reconstitution [50] without adverse effect. Although significant advances have been made in understanding the properties (e.g., physical, chemical, and biological) of so-called smart biomaterials, so far a very limited number of such type of materials have met the demand of clinical need. Nevertheless, the European technology platform reported that smart biomaterials could be key to enabling technology for regenerative medicine and therapeutics [51]. 

In the context of TE, several researchers [52,53,54,55] have demonstrated that smart biomaterials have the ability to maintain and control cellular behavior for functional tissue regeneration. Yuan and coworkers [55] demonstrated that porous ceramic scaffolds alone induce substantial ectopic bone formation without the incorporation of molecules and/or cells. Furthermore, authors have clarified that, in a large bone defect in sheep, micro-structured tricalcium phosphate ceramic had the highest osteoinductive potential for bone tissue repair without the addition of cells and/or growth factors, and they defined such ceramics as a class of smart biomaterials for bone TE.

**Figure 2:**
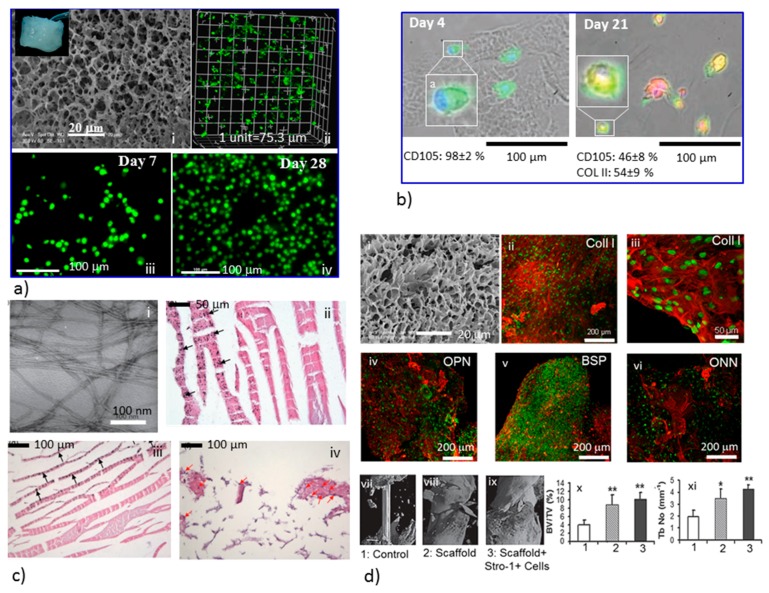
Stimuli-responsive polymeric biomaterials for TE applications: (**a**) CS/PEI, pH responsive hydrogel scaffold, scanning electron microscope image hydrogels frozen in liquid nitrogen and freeze-dried (i), a confocal image of HeLa cells (labeled with CellTracker Green) grown within the hydrogel (day 21) (ii), human fetal skeletal cells (labeled with CellTracker Green) grown within the hydrogel on day 7 (iii) and day 28 (iv); (**b**) influence of photodegradable dynamic microenvironment on chondrogenic differentiation of hMSCs was verified by immunostaining for the hMSC marker CD105 (fluorescein isothiocyanate (FITC), green) and the chondrocyte marker COLII (tetramethyl rhodamine isothiocyanate–labeled, red), cells did not produce COLII on day 4 (left), almost half of the cells with peptide sequence (Arg-Gly-Asp-Ser (RGDS)) strongly expressed CD105, and the other half produced COLII (right) on day 21; (**c**) self-assembly of complementary peptides hydrogels; (i) TEM image of P_11_–13/P_11_–14 peptide fibrils and fibers, prepared at pH 7.4, primary human dermal fibroblasts grown within hydrogel and their histological images P_11_–13/P_11_–14 hydrogel after 14 days of culture (ii,iii). Black arrows indicate possible cell remnants of black, circular aggregates on some fibers. P_11_-4 hydrogel with primary human dermal fibroblasts (red arrows) after 14 days of culture (iv) showing Neo-ECM deposition; (**d**) bioresorbable scaffolds fabricated from polymer blend (CS/Polyvinyl acetate (PVAc)/PLLA: 50/25/25) for bone TE; (i) SEM image of scaffold prepared by freeze drying using a solvent-evaporation technique showing 3D porous network structure, immunostaining for osteogenic bone-matrix proteins of STRO-1 + cells cultured on the scaffold (in vitro), cell nuclei are stained with DAPI (green) and each bone matrix protein is stained by the Alexa 594 fluorochrome-conjugated secondary antibody (red). Confocal microscopic images show Collagen type I (ii, iii), osteopontin (OPN) (iv), bone sialoprotein (BSP) (v) and osteonectin (ONN) (vi). (**d**) (vii–xi) Quantitative μ CT analysis for bone tissue regeneration of selected regions of interest within the osteotomy defect after 28 days. Enhanced bone formation is demonstrated in both scaffold groups (without and with STRO-1 + cells, respectively) when compared to the control group. Assessment of new bone regeneration in the defect regions in femora of mice at 28 days following implantation, using indices of bone volume/total volume (BV/TV) (x) and trabecular number (Tb No) (xi). Results are presented as mean ± SD, *n* = 4 per group, ∗ = *p* < 0.05, ∗∗ = *p* < 0.005. (**a**) reproduced with permission [40]. Copyright 2009, Wiley-VCH Verlag, Germany. (**b**) Reproduced with permission [65]. Copyright 2009, Science. (**c**) Reproduced with permission [47]. Copyright 2012, Wiley-VCH Verlag. (**d**) Reproduced with permission [12]. Copyright 2013, Wiley-VCH Verlag, Germany.

Generally, smart biomaterials respond to one or more environmental variables (e.g., temperature, pH, ionic concentration, light, electric and magnetic fields), which influence cells behavior and functionality as well as tissue modeling. A list of smart biomaterials that relate to cell culture and TE is presented in Table 1 and Figure 1 and Figure 2. A number of research strategies can be adopted for cells attachment, growth, and differentiation towards specific tissue development.

For TE, the biocompatibility—or, no cytotoxicity—is the critical requirement for a smart biomaterial. Successful biocompatibility indicates that such smart materials can be used to develop 3D-specific scaffolds using advanced techniques [17]. Scaffolds can be loaded with specific cell types, proteins, and/or antibiotics, and subsequently implanted for functional tissue development. From a clinical aspect, such smart implants should respond to the variation of their environment in a valuable biological pathway.

For active tissue engineering, smart polymeric biomaterials are being designed for the regulation of stem cell activity and to understand complex cellular processes. Poly(*N*-isopropyl acrylamide) and poly(*N*,*N*′-diacrylamide) (Figure 1b) are common thermo-responsive dynamic polymers with lower critical solution temperature (LCST), which have been explored for cells adhesion, spreading, and release. Light is another stimulus of particular interest, which has been utilized for the degradation of 3D hydrogel networks after stem cell encapsulation to enhance cellular expansion and their chondrogenic differentiation [65], and has proved that the photodegradation of hydrogels networks provide dynamic elastic modulus in a microenvironment very similar to soft tissue, as well as influence the valvular interstitial cell function [68]. Light has also been employed to achieve stronger materials via new crosslinking, resulting in the alteration of material mechanics [69]. In biomaterial scaffolds, dynamic properties can also be introduced by ionic crosslinking such as alginate crosslinking by divalent cations for cells mobility and migration [70], DNA crosslinking for fibroblast remodeling [71], and hydrogen bonding interactions between CS and polyethylenimine for the chondrogenic differentiation of fetal skeletal cells [40].

The control of responsive biomaterials after implantation in the body is more challenging. Hillel and coworkers developed an injectable scaffold for soft tissue replacement using poly(ethylene glycol) (PEG) and hyaluronic acid (HA) that can be crosslinked in situ with light exposure to form a 3D network [72] or modify the characteristic properties of scaffold materials [73]. Ultrasound is another stimulus that has been utilized for ultimate drug delivery in a controlled manner [74]. An example of a non-polymeric trigger, electrochemically activated microchip system, such as a gold membrane, has been developed for controlled release in implanted materials [66].

Shape-memory materials are a subset of stimuli-responsive biomaterials that change their geometry and mechanical properties with the variation of temperature or light [75,76], and may lead to the next generation of dynamic 3D implantable smart biomaterials [77]. Shape-memory alloys (e.g., Nitinol) have already found commercial use in orthodontic, orthopedic vascular, neurosurgical, and surgical fields, and have been well documented [67,78,79]. In the last few decades, a number of polymeric systems have been developed using a variety of functional monomers to generate block, graft, and brush copolymers, along with biodegradable constructs and crosslinked polymers; moreover, some such systems are available for biomedical applications.

Interactions of smart scaffold biomaterials with cells are crucially important, in which cells need to receive signals continuously from biomaterials to regulate cellular function [80]. Smart biomaterials should have the ability to mobilize growth factors in order to modulate cell proliferation and phenotypes (as discussed before). Thus, the surface functionalization of biomaterials can be a key technology to promote the biological performance of smart biomaterials. Smart biomaterials with complex biological functionalities can be achieved either by the physical and/or chemical modification of 3D structural properties to enhance tissue in-growth, vascularization, and nutrient passing [12,81,82]. Furthermore, smart biomaterials could be designed as absorbable scaffolds that are absorbed by the host metabolic activity. However, the design of such absorbable constructs having biodegradation kinetics continuously matching with the progress of new functional tissue development of the host remains challenging. Nevertheless, structural design with sufficient elastic modulus and bioresorption kinetics can be introduced to meet the physical, chemical, and biological properties of implantation that enable minimally-invasive surgery, focusing on repairing that could lead to a more economically viable healthcare system.

Finally, the progress of non-active biomaterials is described. When the TE scaffold comes into contact with cell culture medium or human body fluids, first of all, water molecules adsorb into the TE scaffold, followed by protein adsorptions, deformations, and cell adhesions. The presence of water molecules in TE scaffolds may play a pivotal role in mediating biochemical reactions between cells and scaffold materials. In order to design the appropriate TE scaffold, the water structure and dynamics of the TE scaffold must be considered. There are two types of water structures, namely non-freezing and freezing water, that form the hydrated TE scaffold [83,84,85] (Figure 1d). It has been reported in the published literature [83,84,85] that hydrated biomacromolecules such as DNA, RNA, proteins, and polysaccharides also form intermediate water structures in addition to those of non-freezing and freezing water. The intermediate water is also observed in biocompatible/inert/non-fouling synthetic polymers (Figure 1d) [83,84,85]. The intermediate water dictates the cells behavior such as cellular attachment, proliferation, migration, and differentiation [86,87,88], and can be considered as a potential parameter for designing biomaterial scaffolds.

The molecular structure and dynamics that affect the water structure can be controlled by interactions between the backbone and side chain of polymeric biomaterials [83,89]. Therefore, in theory, the structure and dynamics of polymer molecules can be changed by altering the chain length, the functional groups in its side chain, or the backbone of the polymer. The molecular structure and dynamics of polymers can dictate the intermediate water contents. Using the concept of intermediate water, a biodegradable and biocompatible polymer consisting of an aliphatic carbonyl group has been designed and synthesized by ring-opening polymerization [90]. In the future, we hope to find a novel route and methodology to generate well-defined smart biomaterials by considering chemical, physical, and biological approaches towards the realization of a combinatorial, high-throughput pathway for specific TE applications.

## 5. Concluding Remarks

It is clear smart biomaterials will find many applications in the biomedical field. To date, most of the research has focused on pH- and temperature-responsive smart materials systems as carriers for drugs, antibiotics, proteins, and DNA delivery to the cells. Thermal-responsive smart polymers derived from natural polysaccharides, proteins, synthetic origins, and/or their conjugation and their thermal behavior have been described in conjunction with cell behavior and tissue engineering. The design of smart biomaterials with hierarchy architecture along with stimuli response and healing is crucial. To date, advanced chemical technology provides the knowledge and tools for the synthesis, processing, and characterization of smart polymers with integrated bioactive functionality aiming for biomedical applications. Therefore, the performance and function of such biomaterial systems will eventually depend on the method of assembly and interaction with complex biological interfaces. Although it is not necessary to have all biomimetic properties of a native tissue in smart biomaterials [25], an in-depth understanding of complex biology will facilitate rational design for intended tissue application. To date, the most efficient biomaterials have been achieved from various functional monomers, macromolecules, or oligomers by crosslinking along with biodegradable segments. Such systems are able to interact with cells and promote cell-cell communication in response to stimuli, and are expected to be useful for functional tissue generation, and concurrently act as a delivery agent (e.g., for growth factors, antibiotics, drugs) for faster curing. Self-assembly peptides are excellent biomaterials that respond to physico-chemical stimuli, and promising results have been achieved for 3D cell culture and tissue engineering. However, a number of issues need to be addressed, as this technology in its early stage of development.

Biomaterials after implantation experience a tremendously dynamic environment in physiological complexes that demand better techniques and methodology to monitor biomaterials from a molecular perspective, biodegradation to structural integrity changes, and functional tissue formation. Finally, to facilitate future discovery processes, the rational design of smart biomaterials and their creation in an economically viable route will remain an active field.

## Figures and Tables

**Table 1:** Smart biomaterials for cellular/TE applications that response to various types of stimuli. PEO—polyethylene oxide; PPO—polypropylene oxide; DOX—doxorubicin.

Examples of Smart Biomaterials	External Stimuli	Applications
Poly(*N*-isopropylacrylamide)	Temperature	Patterned cells seeding and co-culture [56].
Pluronics^®^ (poly(ethylene oxide)-poly(propylene oxide)-poly(ethylene oxide))	Temperature	Tissue engineering processes (new cartilage formation [57].
PNIPAm-Arg-Gly-Asp (RGD)	Temperature	Controlling osteoblast adhesion and proliferation [58].
Poly(2-propylacrylic acid)	pH	Protein/DNA intercellular delivery [59].
Chitosan/Polyethyleneimine (CS/PEI) blend	pH	Scaffolds for cellular functioning and cartilage tissue engineering [40].
Self-assembling peptide	Temperature and pH	Neural tissue engineering [46].
Self-assembling peptide	Temperature and pH	Peptide (P_11_-4) supported primary human dermal fibroblasts growth and proliferation [47].
Azobenzene-containing polymer brushes	Light	Human umbilical vein endothelial cells [60].
Spiropyran-containing polymer brushes/graft copolymer	Light	Cell capture and release [61].
Poly(2-acrylamido-2-methyl-propane sulphonic acid-co-*N*-butylmethacrylate)	Electric field	Controlled delivery of drug and cells [62].
Poly(*N*-isopropylacrylamide-acrylamide-chitosan) (PAC)-coated magnetic nanoparticles (MNPs)	Magnetic field, temperature, and pH	Human dermal fibroblasts and normal prostate epithelial cells culture and cancer drug delivery [63].
Poly(6-*O*-methacryloyl-d-galactopyranose)-SS-poly(γ-benzyl-l-glutamate) (PMAgala-SS-PBLG)	Redox reaction	DOX delivery and human hepatoma cell receptor targeting [64].
Poly(ethylene-glycol)-Poly(acrylate)	Light	Human mesenchymal stem cells growth, proliferation, and chondrogenic differentiation [65].
Gold membrane microchip	Electrochemical	Controlled release in implants [66].
Antibacterial Ti-Ni-Cu shape memory alloys	Temperature	Cellular compatible (e.g., L929 and MG63) [67].
